# CaM/BAG5/Hsc70 signaling complex dynamically regulates leaf senescence

**DOI:** 10.1038/srep31889

**Published:** 2016-08-19

**Authors:** Luhua Li, Yangfei Xing, Dong Chang, Shasha Fang, Boyang Cui, Qi Li, Xuejie Wang, Shang Guo, Xue Yang, Shuzhen Men, Yuequan Shen

**Affiliations:** 1 State Key Laboratory of Medicinal Chemical Biology, Nankai University, 94 Weijin Road, Tianjin 300071, China; 2 College of Life Sciences, Nankai University, 94 Weijin Road, Tianjin 300071, China; 3 Synergetic Innovation Center of Chemical Science and Engineering, 94 Weijin Road, Tianjin 300071, China

## Abstract

Calcium signaling plays an essential role in plant cell physiology, and chaperone-mediated protein folding directly regulates plant programmed cell death. The *Arabidopsis thaliana* protein AtBAG5 (Bcl-2-associated athanogene 5) is unique in that it contains both a BAG domain capable of binding Hsc70 (Heat shock cognate protein 70) and a characteristic IQ motif that is specific for Ca^2+^-free CaM (Calmodulin) binding and hence acts as a hub linking calcium signaling and the chaperone system. Here, we determined crystal structures of AtBAG5 alone and in complex with Ca^2+^-free CaM. Structural and biochemical studies revealed that Ca^2+^-free CaM and Hsc70 bind AtBAG5 independently, whereas Ca^2+^-saturated CaM and Hsc70 bind AtBAG5 with negative cooperativity. Further *in vivo* studies confirmed that AtBAG5 localizes to mitochondria and that its overexpression leads to leaf senescence symptoms including decreased chlorophyll retention and massive ROS production in dark-induced plants. Mutants interfering the CaM/AtBAG5/Hsc70 complex formation leads to different phenotype of leaf senescence. Collectively, we propose that the CaM/AtBAG5/Hsc70 signaling complex plays an important role in regulating plant senescence.

Senescence occurs at the final stage of leaf development in plants and is a critical process for the recycling of nutrients from senescent leaves to other parts of the plant. Leaf senescence is regulated by multiple environmental factors including stresses such as drought, nutrient deficiency and temperature, and endogenous factors including aging and phytohormone levels^1^. The process of senescence begins with chloroplast dismantling followed by catabolism of macromolecules such as chlorophyll, proteins and nucleic acids and, subsequently, the degeneration of mitochondria^2^,^3^.

Mitochondrial dysfunction occurs at the late stage of natural and dark-induced leaf senescence, together with the release of cytochrome *c* (cyt *c*) into the cytosol, which results from mitochondrial oxidative burst and mitochondrial transmembrane potential loss^4^. Cyt *c* triggers the activation of caspases^5^, which subsequently causes diminution of the number of mitochondria^6^. Mitochondria serve as an important calcium store in plant cells. Studies have demonstrated that calcium participates in plant senescence by regulating the generation of nitric oxide (NO) because plant NO synthase (NOS) activity is regulated by Ca^2+^/calmodulin signaling^7^,^8^ and/or by modulating the activation of MPK6^9^. Thus, maintaining the balance of the cytosolic calcium ([Ca^2+^]_cyt_) level is particularly important in plant growth and development. Changes in [Ca^2+^]_cyt_ are primarily caused by uptake from extracellular circumstances and/or release from intracellular calcium stores and to a lesser extent by efflux into the extracellular space and/or retrieval into calcium stores^9^,^10^. The specific regulation mechanism of the mitochondrial calcium signaling pathway in the process of plant senescence has not been well characterized.

In plants, calcium signaling is a complicated biological process. Nearly all hormonal, abiotic and biotic stresses can lead to a transient increase in the [Ca^2+^]_cyt_ level. The signal of increasing intracellular Ca^2+^ levels is subsequently sensed by various Ca^2+^-binding proteins, which elicit diverse downstream signaling events^11^. Calmodulin (CaM) is a ubiquitous calcium sensor that is involved in various eukaryotic signal transduction pathways^12^. CaM modulates numerous intracellular responses by regulating the activity of a large number of CaM-binding proteins, including ion channels, protein kinases/phosphatases, transcription factors and metabolic enzymes^13^. Plants possess numerous CaM-binding proteins, many of which are plant specific, suggesting a different and more complicated Ca^2+^/CaM signaling network in plants^14^. CaM consists of two domains (an N- and a C-lobe), each containing two calcium-binding sites. CaM adopts a “closed” conformation in the calcium-free (apo-CaM) state, in which the hydrophobic residues are held together to block the calcium-binding sites, whereas calcium saturation triggers CaM to exhibit an “open” conformation that features extended hydrophobic clefts (holo-CaM)^15^. The transition between the two conformational states triggered by calcium binding provides the structural basis for the recognition of target proteins and downstream calcium signal transduction in response to cellular processes^16^.

The BAG (Bcl-2-associated athanogene) proteins are a multifunctional group of chaperone regulators. As an evolutionarily conserved family, these proteins are distinguished by a common conserved region located near the C-terminus termed the BAG domain (BD) that directly interacts with the ATPase domain of Hsp70/Hsc70. In *Arabidopsis*, there are seven homologs of the BAG protein family, which regulate apoptosis-like processes ranging from pathogen attack and development to abiotic stresses. Based on their characteristic structural components, the *Arabidopsis thaliana* BAG (AtBAG) protein family can be divided into two groups. The first group, comprising four BAG proteins (AtBAG1-4), possesses a ubiquitin-like domain (ULD) at the N-terminus of the BAG domain. In our previous study, we determined the structures of the BAG domains in AtBAG1–4; additional *in vivo* studies indicated that *bag2* plants were larger than the wild type, demonstrating the influence of AtBAG2 on plant growth and development^17^. AtBAG4, which is localized in the cytoplasm, has been demonstrated to be involved in plant tolerance to abiotic stresses such as cold, drought and salt^18^. The second group of AtBAG family members comprises AtBAG5-7. These proteins are characterized by a conserved IQ motif adjacent to the C-terminal BAG domain. The IQ motif is a plant-specific CaM-binding motif with the consensus sequence IQXXXRGXXXR, which preferentially binds to apo-CaM^19^. This novel feature implicates the involvement of BAG proteins in the Ca^2+^/CaM signaling pathway in plants. AtBAG6-induced cell death may be associated with CaM-AtBAG6-mediated cellular responses^20^. AtBAG7, which is localized in the endoplasmic reticulum (ER), has been shown to be involved in the maintenance of the unfolded protein response (UPR)^21^. Here, we identified a signaling complex CaM/AtBAG5/Hsc70 which plays important role in plant senescence.

## Results

### Overall Structures of AtBAG5 Alone and in Complex with Apo-CaM

AtBAG5 contains two protein interaction modules: the IQ motif for CaM binding and the BAG domain for Hsc70 binding. However, little is known regarding how these two modules crosstalk and mediate different signaling outputs. To understand the underlying molecular mechanism, we performed structural and biochemical studies on the interaction of AtBAG5 with CaM and Hsc70.

**Figure 1:**
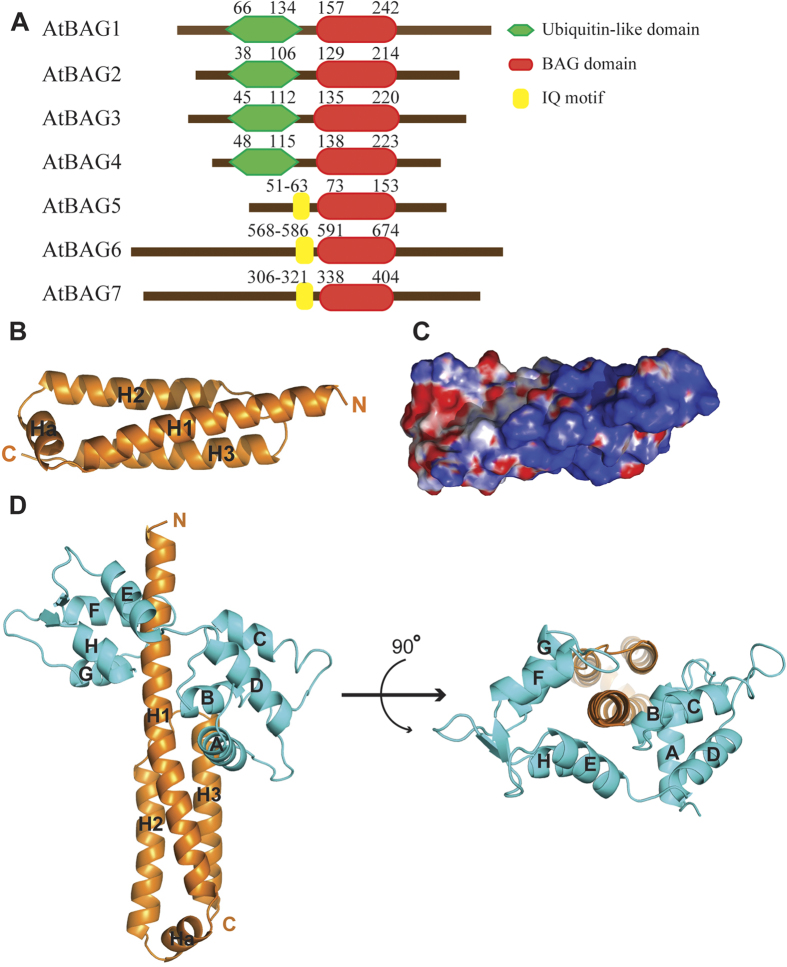
Molecular details of the CaM/AtBAG5/Hsc70 signaling complex. (**A**) Schematic domain organization of the AtBAG5 protein. (**B**) Overall structure of AtBAG5-long. (**C**) The electrostatic surface potential of AtBAG5-long. Red: negatively charged; blue: positively charged; and white: neutral. (**D**) Overall structure of AtBAG5-long (orange) in complex with apo-CaM (cyan). The helices of CaM are designated A–H.

The crystal structure of the AtBAG5 construct (residues 49–153, referred to hereafter as AtBAG5-long), which contains an IQ motif and an adjacent BAG domain (Fig. 1A), was determined at a resolution of 2.0 Å by molecular replacement using our previously determined AtBAG4 structure as the search model^17^. There is one AtBAG5-long molecule in the asymmetric unit. Of the total 105 residues in the AtBAG5-long molecule, only the BAG domain (residues 73–153) is clearly defined. The BAG domain of AtBAG5 is primarily composed of four α-helices (H1, H2, Ha and H3). H1, H2 and H3 form an elongated three-helix bundle, which shares structural similarity with its human homologs^22^. In addition to the short α-helix (Ha) that lies between helices H1 and H2 (Fig. 1B), the BAG domain generally retains the highly conserved helix bundle structure shared by other AtBAG family members^17^. The superposition of AtBAG5 and AtBAG4 gives the root-mean-square deviation value of 1.13 Å for 67 out of 84 Cα atoms (Figure S1A). The presence of the canonical BAG domain suggests the recognition of AtBAG5 by the molecular chaperones Hsp70/Hsc70. Far-Western blot analysis indicated that AtBAG5 and AtHsc70 interact *via* BAG domain binding to the AtHsc70 ATPase domain^20^.

By contrast, the right half of the surface in the structure of AtBAG5-long is primarily positively charged, as indicated by the electrostatic surface potential (Fig. 1C). This region is presumably responsible for the interaction with CaM as CaM exhibits an acidic isoelectric point^23^. Additionally, AtBAG5 contains a predicted IQ motif, which is recognized to be a specific calmodulin-binding site^19^. To investigate the structural basis for the recognition of AtBAG5 by CaM, we determined the structure of the AtBAG5-long/apo-CaM complex. The structure of the complex was determined at 2.5 Å resolution by molecular replacement using the coordinates of AtBAG5-long and apo-CaM as search models, in combination with anomalous scattering signals from Se-Met-substituted apo-CaM. There are two complex molecules in the asymmetric unit. These two molecules are similar, with a root-mean-square deviation of 0.59 Å. In the structure of the complex, the IQ motif is clearly defined, together with helix H1, forming an extended α-helix (Fig. 1D, left panel). The conformation of the BAG domain in the AtBAG5-long/CaM complex is extremely similar to that in free AtBAG5-long (Figure S1B). Except for the poor electron density for the central linker connecting the two lobes, the entire full-length apo-CaM is clearly defined. The four calcium-binding regions of the CaM molecule do not interact with the IQ motif; additionally, electron density corresponding to metal ions is absent in the divalent cation binding sites in CaM. The N-lobe of CaM exhibits the classic closed conformation, whereas the C-lobe adopts a “semi-open” conformation. The F and G helices adjust their orientations to stabilize a comparatively open groove that grips the elongated H1 α-helix of the IQ motif (Fig. 1D, right panel). This CaM conformation is similar to that previously reported for IQ/apo-CaM complexes (Figure S2)^24^,^25^.

### Molecular Recognition of AtBAG5 by Apo-CaM

**Figure 2:**
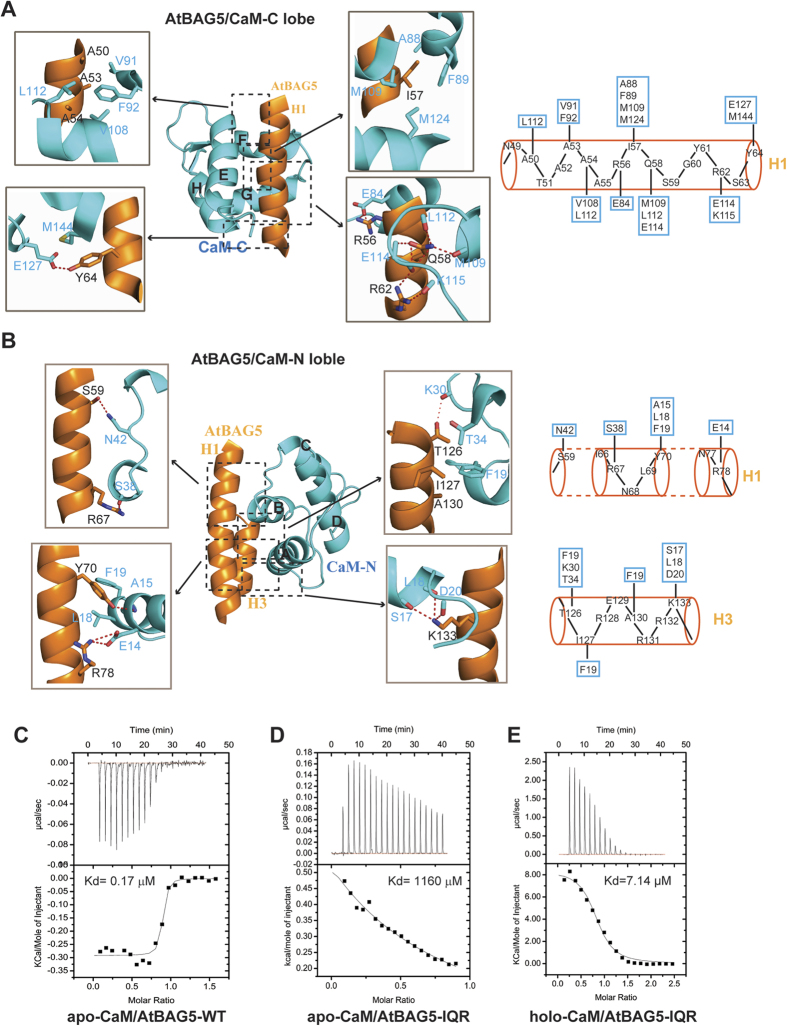
Recognition of AtBAG5 by apo-CaM. (**A**) Detailed interactions of the CaM C-lobe with four regions of the H1 helix in AtBAG5-long. Relevant residues are numbered, and hydrogen bonds are shown as red dashed lines (left panels). Schematic diagram of the major interactions between the CaM C-lobe (cyan) and AtBAG5-long (orange). The CaM C-lobe residues involved in the interactions are boxed (right panel). (**B**) Detailed interactions between the CaM N-lobe and H1 and H3 of AtBAG5-long (left panels). Schematic diagram of the major interactions between the CaM N-lobe and AtBAG5-long (right panels). (**C**–**E**) ITC analysis of the binding affinity of apo-CaM for wild-type AtBAG5-long, apo-CaM for AtBAG5-IQR and holo-CaM for AtBAG5-IQR.

Additional structural analysis revealed the specific interaction between AtBAG5-long and apo-CaM. Specifically, the interaction between the IQ motif and the semi-open C-lobe primarily involves an intensive network of hydrophobic interactions, hydrogen bonds and salt bridges (Fig. 2A). First, the side chain of Ile57 in AtBAG5-long is buried by the hydrophobic cleft formed by the side chains of Ala88, Phe89, Met109 and Met124 in the C-lobe (Fig. 2A, upper right). Second, Arg56 in AtBAG5-long forms a salt bridge with the C-lobe residue Glu84. The side chain of Gln58 in the H1 helix forms essential hydrogen bonds with the main chain atoms of the C-lobe residues Glu114, Met109 and Leu112. Two hydrogen bonds are formed between the side chain of Arg62 in the H1 helix and the main chain carbonyl atoms of the C-lobe residues Glu114 and Lys115 (Fig. 2A, lower right). Third, the side chain of Tyr64 in the H1 helix forms a hydrogen bond with the OE2 atom of Glu127 in the C-lobe and participates in hydrophobic interactions with Met144 in the C-lobe (Fig. 2A, lower left). Fourth, the H1 helix residues Ala50, Ala53 and Ala54 are surrounded by a hydrophobic patch consisting of the C-lobe residues Val91, Phe92, Val108 and Leu112 (Fig. 2A, upper left). These extensive interactions are similar to those in previously determined IQ motif/semi-open C-lobe complex structures^24^,^25^,^26^.

In contrast to the minimal interactions within the N-lobe observed in previously determined IQ peptide/CaM structures^24^,^25^, AtBAG5-long exhibits numerous interactions with the closed N-lobe of CaM (Fig. 2B). Two hydrogen bonds are formed between the OG atom of Ser59 in the H1 helix and the ND2 atom of Asn42 in the N-lobe, in addition to the hydrogen bonds formed between the NH1 atom of Arg67 in the H1 helix and the main chain oxygen of Ser38 in the N-lobe (Fig. 2B, upper left). The side chain of Tyr70 in the H1 helix of AtBAG5-long participates in hydrophobic interactions with the N-lobe residues Ala15, Leu18 and Phe19 and also forms a hydrogen bond with the main chain nitrogen atom of the N-lobe residue Ala15. A salt bridge is formed between Arg78 in the H1 helix and Glu14 in the N-lobe (Fig. 2B, lower left). Additional recognition of AtBAG5-long by the CaM N-lobe includes hydrophobic interactions between Thr126 in the H3 helix and the side chains of the N-lobe residues Phe19 and Thr34. Furthermore, a hydrogen bond is formed between the OG1 atom of Thr126 in the H3 helix and the main chain carbonyl oxygen atom of Lys30 in the N-lobe. Moreover, the H3 helix residues Ile127 and Ala130 participate in hydrophobic interactions with Phe19 in the N-lobe (Fig. 2B, upper right). Lys133 in the H3 helix forms three hydrogen bonds with the main chain carbonyl oxygen atoms of the N-lobe residues Ser17, Leu18 and Asp20 (Fig. 2B, lower right).

To verify the contribution of the IQ motif to the association of AtBAG5-long with apo-CaM, we substituted three highly conserved residues (Ile57, Gln58 and Arg62) in the IQ motif of AtBAG5-long with alanine (a mutant referred to hereafter as AtBAG5-IQR). Isothermal titration calorimetry (ITC) was performed to compare the thermodynamics of apo-CaM binding to AtBAG5-long and AtBAG5-IQR. Upon titration of apo-CaM, wild-type AtBAG5-long exhibited tight association, with a ***K***_d_ value of 0.17 μM, which was approximately 10,000-fold higher than that for AtBAG5-IQR (***K***_d_ = 1,160 μM) (Fig. 2C,D). These results indicate that the recognition of the IQ motif by the semi-open C-lobe of CaM plays a predominant role in the interaction between AtBAG5 and apo-CaM.

### Interactions of AtBAG5 with CaM and Hsc70

**Figure 3:**
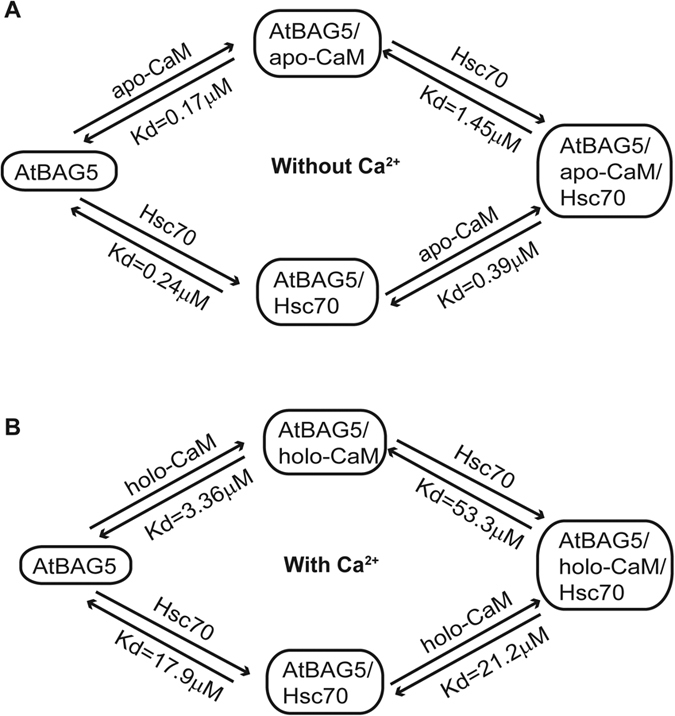
Interactions among CaM, Hsc70 and AtBAG5. (**A**) Schematic diagram shows the relationship among AtBAG5-long, CaM and Hsc70 in the absence of calcium. (**B**) Schematic diagram shows the relationship among AtBAG5-long, CaM and Hsc70 in the presence of calcium.

AtBAG5-long possesses both an IQ motif and a BAG domain, which suggests that it can simultaneously associate with both CaM and Hsc70. Thus, we investigated the binding relationship between AtBAG5 and its two binding partners under different conditions using ITC. In the absence of calcium (Fig. 3A and S3), the binding affinities of AtBAG5-long and the AtBAG5/Hsc70 complex for CaM are similar (0.17 μM versus 0.39 μM). Additionally, the binding affinities of AtBAG5-long and the AtBAG5/apo-CaM complex for Hsc70 are comparable (0.24 μM versus 1.45 μM). These results indicate that in the absence of calcium, AtBAG5-long independently binds CaM and Hsc70. Superposition of the structures of the AtBAG5-long/apo-CaM complex and the AtBAG1/Hsc70 complex reveals different binding sites for apo-CaM and Hsc70 in AtBAG5-long that do not overlap (Figure S1C).

Unexpectedly, in the presence of calcium, calcium-saturated CaM (holo-CaM) is able to bind the AtBAG5-IQR mutant with high affinity (***K***_d_ = 7.14 μM), indicating that the binding mode of AtBAG5 for holo-CaM differs from that for apo-CaM (Fig. 2E). Furthermore, the AtBAG5/Hsc70 complex exhibits an approximately 50-fold reduced affinity for holo-CaM than for apo-CaM (Fig. 3A,B), suggesting that holo-CaM and Hsc70 may bind AtBAG5 with negative cooperativity. ITC analysis indicates that AtBAG5 and the AtBAG5/Hsc70 complex bind holo-CaM *via* different modes (3.36 μM versus 21.2 μM) (Fig. 3B and S3). Therefore, it is likely that following calcium loading, CaM alters its binding mode to AtBAG5, which further attenuates Hsc70 binding to AtBAG5. A negative correlation exists between holo-CaM and Hsc70 in their interactions with AtBAG5. Apo-CaM and holo-CaM binding to different sites on a protein has been proposed for the Na_V_1.2 voltage-dependent sodium channel^25^.

### *AtBAG5* Overexpression Accelerates Dark-induced Leaf Senescence

**Figure 4:**
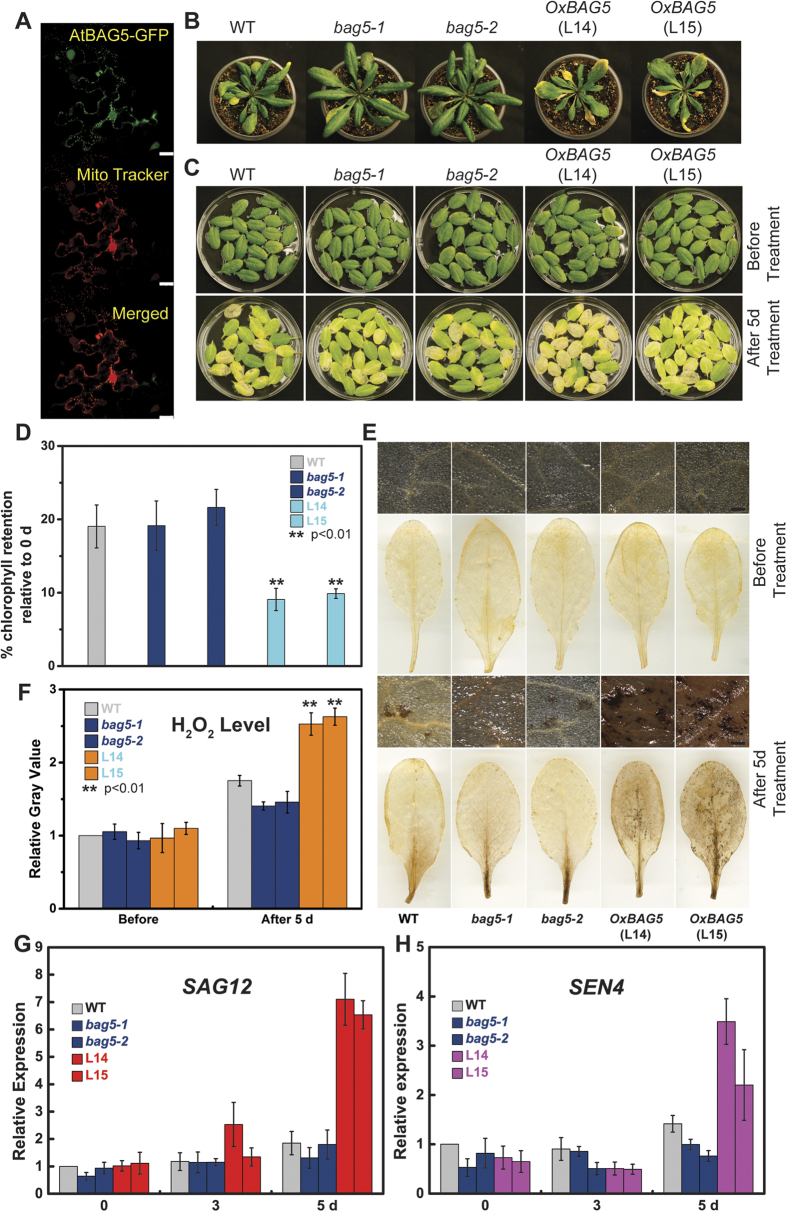
Effect of AtBAG5 on leaf senescence. (**A**) Co-localization experiment using confocal microscopy. AtBAG5-EGFP-TerAtBAG5 fluorescence and CD3-991 fluorescence are shown in green and red, respectively. Yellow indicates colocalization of the BAG5-EGFP and marker signals. Scale bar = 25 μm. (**B**) Senescence phenotype of wild type, *bag5-1*/*bag5-2* mutants and *OxBAG5* transgenic plants. Rosettes from 38-day-old plants are shown. (**C**) Leave phenotype of wild type, *bag5-1*/*bag5-2* mutants and *OxBAG5* plants before and after 5-day dark treatment. The experiment was performed with ≥3 replicates and ≥10 plants in each replicate. (**D**) The percentage of chlorophyll retention was calculated relative to day 0. The experiment was performed with ≥3 replicates. (**E**) DAB staining of detached leaves without treatment and treated with 5-day dark treatment. The results were reproduced in three independent experiments using three plants in each experiment. Scale bar = 500 μm. (**F**) Quantitative gray values of ROS production were measured from DAB stained leaves. (**G**,**H**) The expression levels of *SAG12* and *SEN4* in the wild type, *bag5-1*/*bag5-2* mutants, and *OxBAG5* overexpression lines. Values are mean ± SE (n = 3 experiments). The relative expression was normalized using the internal control *TIP41*.

To further understand the physiological function of the signaling complex CaM/AtBAG5/Hsc70, we take advantage of Arabidopsis thaliana system. At first, we studied the localization of AtBAG5 protein. Based on the prediction from the PSORT program, AtBAG5 exhibits a unique mitochondrial localization^18^. To confirm whether AtBAG5 localizes to the mitochondria, we performed colocalization experiments using a mitochondria-specific dye CD3-991. AtBAG5-EGFP and CD3-991 were co-transfected into *Nicotiana benthamiana* leaf epidermal cells, and the fluorescence expression of GFP and mCherry dye were visualized using confocal microscopy (Fig. 4A). The AtBAG5-EGFP signal colocalized well with CD3-991, suggesting that AtBAG5 localizes to mitochondria.

To delineate the physiological role of *Arabidopsis* BAG5, T-DNA insertion alleles for *AtBAG5*, SALKseq_037369 and SALK_205784C (designated *bag5-1* and *bag5-2*, respectively) were obtained from the Arabidopsis Biological Resource Center (ABRC). The T-DNA insertion flanking sequence revealed that *bag5-1*/*bag5-2* possessed an identical T-DNA insertion site at base pair 594 from ATG (Figure S4A). RT-PCR analysis indicated that *AtBAG5* transcription was not detected in the *bag5-1*/*bag5-2* mutants (Figure S4B). Both *bag5* mutant alleles did not exhibit growth defects (Figure S4C and S4D). Quantification of the seedling root length and hypocotyl length indicated that the *bag5-1*/*bag5-2* mutants possessed a shorter root length than the wild type (Figure S4E and S4F). However, the rosette size of 4-week-old *bag5-1*/*bag5-2* mutant plants did not exhibit differences compared with the wild type (Figure S4G). At maturation, *bag5-1*/*bag5-2* mutant plants exhibited phenotypes of delayed senescence (Fig. 4B).

To analyze the functions of AtBAG5 on plant growth and development, we also generated *AtBAG5* overexpression lines (*OxBAG5*). The overexpression of *AtBAG5* was verified through RT-PCR and qRT-PCR (Figure S5A and S5B). In contrast to the *bag5* mutant plants, *OxBAG5* transgenic plants exhibited early senescence phenotypes (Fig. 4B). These results indicate that the expression level of *AtBAG5* correlates with plant senescence.

The effect of AtBAG5 was further investigated in dark-induced leaf senescence. Compared to other external leaf senescence-inducing stimuli, dark-induced leaf senescence can produce synchronous senescence and is commonly used to induce senescence^27^. After a 6-day dark treatment of intact plants, we compared the phenotypes of the wild type, *bag5* mutants, and *OxBAG5* transgenic plants. We found that nearly all leaves turned yellow in *OxBAG5* transgenic plants, whereas more leaves remained green in *bag5-1*/*bag5-2* mutants than in the wild type (Figure S6A). After 3 days of light recovery, *OxBAG5* transgenic plants exhibited the slowest recovery, whereas the *bag5-1*/*bag5-2* mutants returned more rapidly to normal growth and had less dead leaves (Figure S6A).

Subsequently, we performed a dark-induced leaf senescence assay in detached leaves from 30-day-old plants. Leaves were photographed, and the chlorophyll content was quantified on day 0 and following a 5-day dark treatment. After a 5-day treatment, nearly all detached leaves of *OxBAG*5 transgenic plants exhibited severe yellowing, wild-type plants possessed less yellow leaves, and *bag5-1*/*bag5-2* mutants possessed the most leaves that remained green (Fig. 4C). The chlorophyll content was consistent with these phenotypes (Fig. 4D).

In *Arabidopsis*, reactive oxygen species (ROS) are considered signaling molecules in leaf senescence^28^,^29^. Dark-induced senescence is often accompanied by the accumulation of H_2_O_2_^30^. To further understand the role of AtBAG5 in leaf senescence, we examined H_2_O_2_ levels in dark-treated leaves of the wild type, *ba*g5 mutants and *OxBAG5* plants. Fresh leaves of 30-day-old plants were used for DAB staining. We found that the basal level of H_2_O_2_ is similar in leaves from different genotypes (Fig. 4E). However, following dark treatment, leaves from *OxBAG5* transgenic lines exhibit a greater accumulation of H_2_O_2_ than leaves from the wild type or *bag5-1*/*bag5-2* mutants (Fig. 4E,F). These results suggested that AtBAG5 participates in leaf senescence by regulating the production of ROS. The up-regulated expression of AtBAG5 occurs with the accumulation of ROS, which further accelerates the process of leaf senescence.

Leaf senescence is often accompanied by the up-regulated expression of certain genes. These genes, which are defined as senescence-associated genes (SAGs), precisely control the progress of leaf senescence. Previous studies have confirmed that the genes *SAG12*, *SAG20* and *SEN4* are upregulated during leaf senescence^31^,^32^,^33^. In our study, RNA was extracted from detached leaves, and the transcript levels of these three SAGs were detected using qPCR. After 3-day and 5-day dark treatments, the transcript levels of these genes were found to be substantially higher in *OxBAG5* transgenic leaves than those in *bag5-1*/*bag5-2* and wild-type leaves. In *bag5-1*/*bag5-2* leaves, the transcript levels of *SAG12* and *SEN4* exhibited down-regulated expression compared with those in the wild-type leaves (Fig. 4G,H), whereas *SAG20* exhibited a transcript level that was similar to that in the wild type (Figure S6B). Collectively, we conclude that AtBAG5 participates in leaf senescence not only through the regulation of ROS production but also through the modulation of SAG expression.

### CaM/AtBAG5/Hsc70 Signaling Pathway is Important for Leaf Senescence

**Figure 5:**
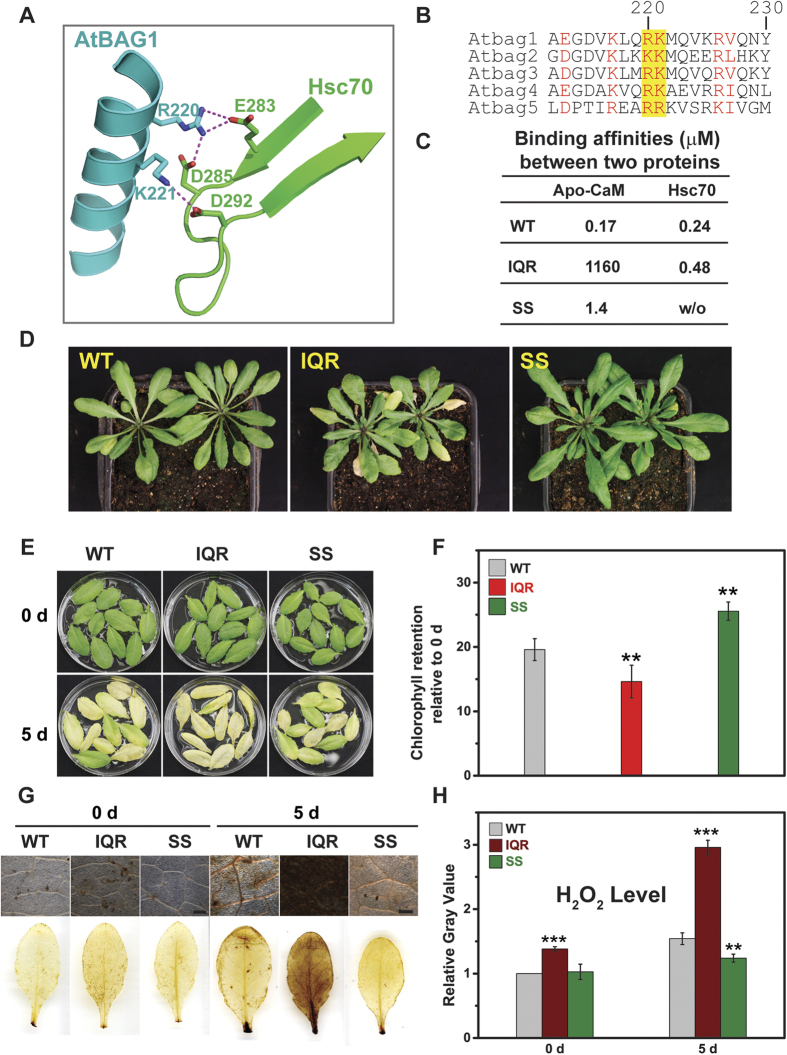
Characteristics of gain- and loss-of-function AtBAG5 mutant plants. (**A**) The detailed interaction of AtBAG1 (cyan) with Hsc70 (green). (**B**) Sequence alignment of AtBAG1-AtBAG5. Conserved residues are colored red. Key residues responsible for the association of the BAG domain with Hsc70 are highlighted yellow. (**C**) Binding affinities of wild-type and mutant AtBAG5 with CaM and Hsc70. w/o indicates no detectable association with ITC. (**D**) Senescence phenotypes of wild type, IQR mutant and SS mutant. (**E**) Leaf phenotypes of wild-type, IQR mutant and SS mutant plants before and after 5-day dark treatment. (**F**) The percentage of chlorophyll retention was calculated relative to day 0. **Denotes p < 0.05. (**G**) DAB staining of detached leaves without treatment and after 5 -day dark treatment. Scale bar = 500 μm. (**H**) Quantitative gray values of ROS production were measured. **Denotes p < 0.05; ***denotes p < 0.01.

To further investigate the physiological role of CaM/AtBAG5/Hsc70, two mutants were constructed to disrupt the association of either CaM with AtBAG5/Hsc70 or CaM/AtBAG5 with Hsc70. According to a previously reported structure of the AtBAG1/Hsc70 complex, residues Arg220 and Lys221 of AtBAG1 form salt bridges with several acidic residues of Hsc70 (Fig. 5A). Aligning the sequences of AtBAG1 andAtBAG5 revealed that residues Arg131 and Arg132 are potentially responsible for the association of AtBAG5 with Hsc70 (Fig. 5B). Indeed, mutating both Arg131 and Arg132 to serines (named SS hereafter) abolished the binding between AtBAG5 and Hsc70 while preserving the association of CaM with AtBAG5 (Fig. 5C). In contrast, the IQ motif mutant in which residues Ile57, Gln58 and Arg62 were mutated to alanines (named IQR hereafter) preserves the association of AtBAG5 with Hsc70 while disrupting the association of AtBAG5 with apo-CaM (Fig. 5C).

We then generated *IQR* and *SS* mutant-overexpression lines. Compared with the wild type, *IQR* mutant plants show early leaf senescence symptoms, whereas *SS* mutant plants show delayed leaf senescence (Fig. 5D). Subsequently, dark-induced leaf senescence assays were performed. Detached leaves from *IQR* transgenic plants exhibited more severe yellowing, while leaves from *SS* transgenic plants showed much less yellowing (Fig. 5E). The quantitative percentage of chlorophyll retention was consistent with these phenotypes (Fig. 5F).

Moreover, H_2_O_2_ levels were examined in dark-treated leaves of from wild-type, *IQR* and *SS* plants. After a 5-day dark treatment, leaves from *IQR* transgenic lines exhibited higher H_2_O_2_ accumulation compared with leaves from the wild type, whereas leaves from *SS* transgenic lines showed much lower H_2_O_2_ levels than those in the wild type (Fig. 5G,H). Taken together, our data suggested that the release of Hsc70 from the CaM/AtBAG5/Hsc70 triple complex favors delayed leaf senescence.

## Discussion

In this study, we demonstrate that AtBAG5 acts as a signaling hub that links the Ca^2+^-signaling network with the Hsc70 chaperone system to regulate plant senescence.

### AtBAG5 in Leaf Senescence

AtBAG5 is quite unique in possessing two classical protein interaction modules: an IQ motif to bind with apo-CaM and a BAG domain to bind with Hsc70. The interaction of AtBAG5 with Hsc70 was demonstrated by the crystal structure of AtBAG5 alone, which reveals the conserved BAG domain, suggesting a conserved binding behavior to Hsc70 as previously described for the AtBAG1/Hsc70 complex^17^. In plants, AtBAG proteins have been demonstrated to function as Hsc70/Hsp70 nucleotide-exchange factors^34^, and increased levels of Hsc70/Hsp70 correlated with a decrease in plant programmed cell death (PCD) caused by abiotic stress^35^.

The interaction of AtBAG5 with apo-CaM was revealed by a crystal structure of the AtBAG5/apo-CaM complex. Structural and biochemical evidence indicated that AtBAG5 is able to bind apo-CaM and Hsc70 simultaneously and independently. Unexpectedly, Ca^2+^-bound CaM most likely alters its binding mode to AtBAG5, which may further attenuate AtBAG5 binding to Hsc70. This mechanism also accounts for the competitive relationship between CaM and Hsc70 in binding to AtBAG5 in the presence of Ca^2+^, indicating that Ca^2+^ up-regulation can induce the release of Hsc70 by disrupting AtBAG5/Hsc70 binding. Therefore, AtBAG5 acts as a molecular hub that connects CaM and Hsc70 in order to sense elevated Ca^2+^ signals and release the Hsc70 molecule.

To investigate the physiological role of CaM/AtBAG5/Hsc70 complex, we first show that the AtBAG5 protein is localized to mitochondria in plant cell. A series of *in vivo* studies revealed that AtBAG5 regulates leaf senescence by controlling the production of ROS and the expression of senescence-associated genes. *OxBAG5* transgenic plants (AtBAG5 overexpression) exhibited early senescence symptoms in both age-dependent and dark-induced leaf senescence. Conversely, *bag5-1*/*bag5-2* plants (AtBAG5 deficient) exhibited delayed leaf senescence. Furthermore, compared with the wild type and *bag5-1*/*bag5-2* mutants, high levels of ROS accumulation and up-regulated senescence-associated gene expression were detected in *OxBAG5* transgenic plants. Thus, we concluded that AtBAG5 is capable of regulating plant senescence.

In animal cells, Hsp70/Hsc70 is already known to play an important role in antiapoptotic function by either inhibiting the formation of apoptosomes^36^ or by inhibiting the release of apoptosis-inducing factor from mitochondria into the cytosol^37^,^38^. In plant, the overexpression of mitochondrial heat shock protein 70 (mtHsp70/mtHsc70) suppressed PCD in rice protoplasts by inhibiting the amplification of ROS^39^. Our results are consistent with these findings. We showed that AtBAG5 mainly localized inside mitochondria and overexpressing AtBAG5 in mitochondria leads to increased ROS levels, which are presumably caused by decreased free Hsc70 molecule due to tight association of AtBAG5 with Hsc70. Further gain-of-function SS mutant plants, which were designed to disrupt the AtBAG5/Hsc70 interaction, showed delayed leaf senescence, whereas loss-of-function IQR mutant plants designed to disrupt the interaction of AtBAG5 with CaM showed early leaf senescence. Therefore, the release of the Hsc70 molecule from CaM/AtBAG5/Hsc70 signaling complex seems to determine the degree of leaf senescence. These results strongly suggest that the CaM/AtBAG5/Hsc70 signaling complex plays a direct role in regulating leaf senescence.

### Proposed Model for CaM/AtBAG5/Hsc70 signaling to Regulate Leaf Senescence

Mitochondria are found to closely associate with Ca^2+^ signaling^40^. By acting as the source of ATP energy and the intracellular calcium pool, mitochondria perform diverse cellular functions in plant growth and development. Mitochondria have been reported to be involved in plant cell death through the regulation of [Ca^2+^]_cyt_ homeostasis and ROS generation^41^,^42^. An elevation in ROS and the expression of senescence-associated genes have been found to be closely related to leaf senescence^43^,^44^. Our results suggest that mitochondrial Ca^2+^ can induce the release of Hsp70 by disrupting AtBAG5/Hsp70 binding. The novel correlation of Hsp70 with the calcium level mediated by AtBAG5 eventually leads to the inhibition of PCD progression and prevents the process of leaf senescence.

**Figure 6:**
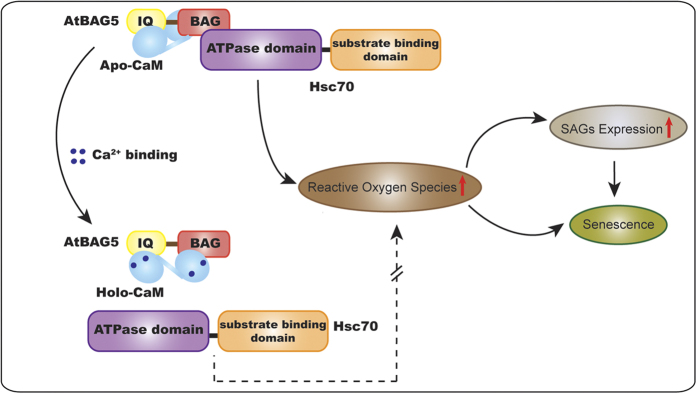
Proposed model of mitochondrial Ca2+ regulation of leaf senescence. At low calcium levels, apo-CaM and the ATPase domain of Hsc70 independently bind AtBAG5. The AtBAG5/Hsc70 complex promotes ROS production and SAG transcription, leading to accelerated leaf senescence. At high calcium levels, holo-CaM alters its binding mode to AtBAG5, which disrupts Hsc70 binding. The release of Hsc70 inhibits the amplification of ROS and further inhibits the senescence pathway.

Based on our results, we propose a model for the influence of Ca^2+^ on the physiological role of AtBAG5 in leaf senescence (Fig. 6). AtBAG5 plays a regulatory role in leaf senescence through the Hsc70-mediated signaling pathway. In addition, CaM can regulate the activity of its binding partners by sensing changes in the calcium level. Apo-CaM binds to the IQ motif on AtBAG5, and their interaction does not affect Hsc70 binding to the BAG domain. Upon elevation of the calcium concentration, CaM alters its interaction mode with AtBAG5. This distinct binding mode to AtBAG5 can further disrupt Hsc70 binding to the BAG domain, leading to the release of Hsc70 from the BAG domain. Free Hsc70 correlates with a decrease in plant PCD by inhibiting the amplification of ROS^35^,^39^. Thus, the crosstalk between the Hsp70 chaperone system and the calcium signal mediated by AtBAG5 eventually leads to inhibition of the senescence pathway.

CaM is an important Ca^2+^ sensor in all eukaryotic cells, binding numerous targets to regulate different cellular processes. However, that CaM is inside mitochondria has been controversial for more than decades. In the earlier studies, the presence and localization of mitochondrial calmodulin was reported using immuno-electron microscopy in animals^45^ and in plant^46^. Additionally, classical CaM-interacting proteins, including Arabidopsis Phosphodiesterase 2A^47^ and Arabidopsis ATPase family gene 1-like protein 1^48^ have been found in plant mitochondria, implying that CaM or CaM-like proteins may also exist in mitochondria. Furthermore, the *Arabidopsis* genome encodes numerous CaM and CaM-like proteins^49^. Among them, CaM-like protein 3 and CaM-like protein 30 were found in mitochondria^50^. The characteristics of these two proteins are very similar to the classical CaM molecule. Therefore, it is likely that either CaM or CaM-like proteins are directly involved in mitochondrial Ca^2+^ signaling. Alternatively, CaM or CaM-like proteins may be transported inside mitochondria to regulate Ca^2+^ signaling pathway. Hsc70 and BAG5 are highly conserved, and their localization inside mitochondria has been confirmed^51^,^52^. In all, these data suggest that the CaM/BAG5/Hsc70 complex is presumably able to transduce calcium signaling to the chaperone system. The exact localization of CaM/AtBAG5/Hsc70 awaits further elucidation in the future. Nevertheless, the signaling pathway mediated by CaM/AtBAG5/Hsc70 provide a novel path to investigate the mechanism of plant senescence.

## Methods

### Plant Materials and Plant Growth Conditions

T-DNA insertion mutants *bag5-1* (SALK_037369) and *bag5-2* (SALK_205784C) in the Columbia (Col-0) background were obtained from the Arabidopsis Biological Resource Center (ABRC) (http://www.Arabidopsis.org). The T-DNA insertion was confirmed using PCR, and *BAG5* transcription levels were determined using semi-quantitative RT-PCR and quantitative RT-PCR. Seeds were surface sterilized with 70% [v/v] ethanol for 5 min and then 1% [v/v] sodium hypochlorite for 10 min, followed by washing three times with distilled water for 5 min each time. Seeds were stratified at 4 °C for 3 days in the dark. Seedlings were grown vertically on MS medium. One-week-old plants were transferred to soil. The plants were grown at 22 °C under long-day conditions (a 16-h-light/8-h-dark cycle).

### Chlorophyll Assays

The 5th and 6th (cotyledons were excluded) leaves were harvested from 30-day-old soil-grown plants. The leaves were weighed, and chlorophyll was extracted on day 0 (untreated) and after a 5-day dark treatment. Extraction and quantification of chlorophyll were performed according to previously described protocols^33^,^53^. Chlorophyll was extracted by placing the tissues in 90% ethanol at 65 °C for 3.5 h several times with gentle blending to confirm that all tissues became chlorophyll free. Subsequently, the solution was cooled to room temperature. Total chlorophyll was determined by measuring the absorbance at 664 and 647 nm using a microplate reader (EnSpire®) and calculated using the following formula: micromoles of chlorophyll per milliliter per gram fresh weight = 7.93(A_664_) + 19.53(A_647_).

### Leaf Senescence Assays

For the whole-plant senescence assay, 24-day-old plants were transferred to a completely dark box in the growth room. Plants were treated for 6 days in the dark and then returned to light for 3 days to recover. Images were taken after 0 days (before treatment), 6 days (after treatment) and 9 days (three days of recovery).

For the detached-leaf senescence assay, the 5th and 6th rosette leaves (cotyledons were excluded) of 30-day-old plants were cut and placed into a plate containing 25 ml of 3 mM MES (pH 5.7) with or without 20 mM calcium chloride dihydrate (CaCl_2_.2H_2_O) at 22 °C in the dark. Images were taken after 0, 3 and 5 days.

### Analysis of H_2_O_2_ Accumulation

The accumulation of H_2_O_2_ was detected by 3,3′-diaminobenzidine (DAB) staining^54^. Detached 5th and 6th leaves of 30-day-old plants after a 5-day dark-treatment with or without 20 mM CaCl_2_ were immersed in DAB solution (1 mg/ml; pH 3.8) overnight to detect *in situ* accumulation of H_2_O_2_. Leaves were fixed in 100% ethanol for 3 h and then boiled in 95% ethanol for 10 min to remove chlorophyll prior to imaging.

### AtBAG5 protein localization

The co-localization assays of ProAtBAG5:AtBAG5-EGFP-TerAtBAG5 with mitochondria marker (CD3-991) was carried out in *Nicotiana benthamiana*. Conditions of *N. benthamiana* plants growth and transformation of Agrobacterium tumefaciens were performed according to previously described protocol^55^. The co-localization was detected using confocal microscopy.

### Protein Expression and Purification

AtBAG5-long, the AtBAG5-IQR mutant, CaM and the ATPase domain of human Hsp70 were expressed in *Escherichia coli* and purified using chromatography techniques. For details, please refer to the Supplemental Methods.

### Structure Determination

AtBAG5-long alone and in complex with CaM was crystallized at 20 °C. All data were collected at the BL17U1 beamline at the Shanghai Synchrotron Radiation Facility (SSRF). The structures were determined by molecular replacement and refined. The represented electron density maps of AtBAG5-long alone and in complex with CaM were shown in Figure S1D and S1E. For details, please refer to the Supplemental Methods.

### Isothermal Titration Calorimetry (ITC) Experiments

ITC measurements were performed using an iTC200 microcalorimeter (MicroCal) in buffer T_20_N_500_ (20 mM Tris-HCl, pH 7.5, and 500 mM NaCl) at room temperature. For the experiments involving apo-CaM, 5 mM EGTA was added to the experimental buffer and all protein samples. Prior to experiments, individual CaM, Hsp70, and complexes of AtBAG5-long/Hsp70 or AtBAG5-long/apo-CaM were eluted from a Superdex 200 10/300 GL size exclusion column (GE Healthcare) with sizing buffer T_20_N_500_ containing 5 mM EGTA. For the experiments involving holo-CaM, all protein samples were eluted from the Superdex 200 10/300 GL size exclusion column with running buffer T_20_N_500_ containing 10 mM CaCl_2_. All samples were centrifuged at 15,000 × g to degas the samples, and the concentrations were determined using both Bradford assay and spectrophotometry (at 280 nm). Sample solutions with a concentration of 0.05–0.1 mM were placed into the sample cell, and the titration solution in the injection syringe was maintained at approximately 0.5–1.0 mM. To determine the binding constants, 20 consecutive injections of the titration solution into the calorimeter cell were collected at 120 intervals with stirring at 1,000 rpm. The titration data were analyzed using MicroCal Origin software (MicroCal).

## Additional Information

**How to cite this article**: Li, L. *et al.* CaM/BAG5/Hsc70 signaling complex dynamically regulates leaf senescence. *Sci. Rep.* **6**, 31889; doi: 10.1038/srep31889 (2016).

## Supplementary Material

Supplementary Information
