# Supplementary material for: CaM/BAG5/Hsc70 signaling complex dynamically regulates leaf senescence
Source: Sci Rep. 2016 Aug 19;6:31889. doi: 10.1038/srep31889 (PMC4990970; doi:10.1038/srep31889)
Supplement: Supplementary Information [file srep31889-s1.pdf]

Supplemental Information

CaM/BAG5/Hsc70 signaling complex dynamically regulate leaf senescence

Luhua Li[1,2], Yangfei Xing[1,2], Dong Chang[1,2], Shasha Fang[2], Boyang Cui[2], Qi Li[2], Xuejie Wang[2], Shang Guo[1], Xue Yang[1,4], Shuzhen Men[2,4], Yuequan Shen[1,2,3,4]

[1]State Key Laboratory of Medicinal Chemical Biology, Nankai University, 94 Weijin Road, Tianjin 300071, China

[2]College of Life Sciences, Nankai University, 94 Weijin Road, Tianjin 300071, China

[3]Synergetic Innovation Center of Chemical Science and Engineering, 94 Weijin Road, Tianjin 300071, China

[4]To whom correspondence may be addressed.

Xue Yang,

E-mail: yangxue@nankai.edu.cn; Tel: +86-22-85358251

Shuzhen Men,

E-mail: shuzhenmen@nankai.edu.cn; Tel:+86-22-23500856

Yuequan Shen,

E-mail: yuequan74@yahoo.com; yshen@nankai.edu.cn; Tel: +86-22-23506462

Supplemental Methods

Supplemental Table SI Data collection and refinement statistics for AtBAG5 structures.

Supplemental Figure S1 Structure of AtBAG5.

Supplemental Figure S2 Superposition of the AtBAG5-long/apo-CaM complex with three reported IQ/apo-CaM complexes.

Supplemental Figure S3 ITC analysis of the binding affinities among CaM, Hsc70 and AtBAG5-long.

Supplemental Figure S4 Isolation and characterization of the bag5-1 and bag5-2 mutants.

Supplemental Figure S5 Analysis of AtBAG5 expression levels in OxBAG5 transgenic plants.

Supplemental Figure S6 Effect of AtBAG5 overexpression on leaf senescence.

Supplemental Methods

Gene Expression Analysis

For semi-quantitative RT-PCR, total RNA was extracted using TRIzol reagent (Invitrogen) and treated with RNase-free DNase I (Fermentas) according to the manufacturer's instructions. Complementary DNA (cDNA) was produced using 1 μg of total RNA and TransScript First-Strand cDNA Synthesis SuperMix from TransGen Biotech (Code#AE301-02) according to the manufacturer's instructions. Semi-quantitative RT-PCR was performed using 2 μl of cDNA samples. *Actin2* was used as an internal control to quantify the relative transcript levels of the target genes.

For real-time RT-PCR detection, RNA extraction and transcription were performed as previously described. cDNA samples were diluted tenfold, and 2 μl was used as the template. The reaction was performed using 2 μl of SYBR Green Perfect mix (TaKaRa, Dalian, China) and a CFX96 Real-Time PCR Detection System (Bio-Rad). The *Tip41* transcript was used as an internal control to quantify the relative transcript levels of the target genes. Each of the three biological replicates was examined in triplicate.

To generate the overexpression transgenic plants, the full-length cDNA of *BAG5* was cloned into the pBA002 binary vector in the sense orientation downstream of the CaMV 35S promoter.

To generate the overexpression of *BAG5* point mutation transgenic plants, the full-length cDNA of AtBAG5 with specific point mutants in BAG domain (SS) and IQ motif (IQR) was obtained, respectively. Then the fragments were cloned into the

pBA002 binary vector in the sense orientation downstream of the CaMV 35S promoter.

All constructs were confirmed via DNA sequencing. The recombinant plasmid was introduced into wild-type *Arabidopsis* using the floral-dip method [1]. Seeds were harvested and sown in soil, and Basta screening was performed. Homozygous T3 plants with single-insert were used for further analysis.

Agrobacterial Infiltration

One-month-old plants of *Nicotiana benthamiana* were used for agrobacterial infiltration. *Agrobacterium tumefaciens* cells (C58C1) containing the ProAtBAG5:BAG5-EGFP construct and the mitochondrial marker (CD3-991), respectively were cultured to OD_{600} = 0.6, then the bacteria were pelleted by centrifugation at 5,000 rpm, resuspended in infiltration buffer (10 mM $MgCl_2$, 10 mM MES, pH 5.9, and 150 μM acetosyringone) to OD_{600} = 1, and incubated at room temperature for 3 h before infiltration. The agroinfiltrated plants were allowed to grown in growth chambers for 48 h before examined by confocal laser-scanning microscopy.

Protein Expression and Purification

Gene fragment corresponding to AtBAG5-long (residues 49-153) was PCR amplified from the *Arabidopsis thaliana* cDNAs and then cloned into the pET-M vector (Novagen). Mutant AtBAG5-IQR was created using a standard PCR-based mutagenesis method and confirmed by DNA sequencing. *Escherichia coli* BL21 (DE3) Codon-Plus cells harboring the recombinant expression plasmid were incubated at

310 K to an OD_{600} of 0.6 and then induced with 0.2 mM isopropyl-β-D-thiogalactopyranoside (IPTG) at 298 K for 16-18 hours. *E.coli* cells were resuspended in buffer $T_{20}N_{200}I_{10}$ (20 mM Tris-HCl, pH 7.5; 200 mM NaCl and 10 mM imidazole) containing 1 mM phenylmethylsulfonyl fluoride (PMSF) before cell lysis using sonication. After the lysate was spun at 18,000 rpm for 30 min, the supernatant was loaded directly onto a Ni-NTA agarose column (Qiagen) which was equilibrated with buffer $T_{20}N_{200}I_{10}$. After washing the Ni-NTA column with 5 column volumes of equilibrium buffer, the 6×His-tagged protein was eluted with buffer $T_{20}N_{200}I_{300}$ (20 mM Tris-HCl, pH 7.5; 200 mM NaCl and 300 mM imidazole). The eluted protein was then loaded onto a HiPrep 26/10 Desalting column (GE Healthcare) to change into buffer $T_{20}N_{200}$ (20 mM Tris-HCl, pH 7.5; 200 mM NaCl). After digestion with PreScission protease to remove His tag, the protein mixture was loaded onto a Ni-NTA agarose column pre-equilibrated with buffer $T_{20}N_{200}$ (pH 7.5) containing 1 mM PMSF to remove the small amount of contaminant protein. The final target protein was loaded onto a HighLoad 26/60 Superdex-200 size-exclusion column (GE Healthcare) and eluted with $T_{20}N_{200}E_1D_1$ (20 mM Tris-HCl, pH 7.5; 200 mM NaCl; 1 mM EDTA; and 1 mM DTT) at a flow rate of 2.0 ml/min. Each fraction of the column elute was 4 ml. The elution peak was identified on SDS-PAGE gel, then the protein was pooled together followed by concentration using Centricon (Millipore).

Human CaM was expressed in bacteria and purified as described previously [2,3]. The AtBAG5-long/apo-CaM complex was formed by mixing the separately purified

proteins together and then subjected to the HighLoad 26/60 Superdex-200 size-exclusion column (GE Healthcare) and eluted with buffer $T_{20}N_{500}E_5D_1$ (20 mM Tris-HCl, pH 7.5; 500 mM NaCl; 5 mM EGTA and 1 mM DTT).

The Se-Met derivative protein of CaM was produced following the same protocol as that of the wild-type protein, with the exception that methionine auxotroph *E.coli* B834 (DE3) cells and minimal medium were used.

The ATPase domain of human Hsc70 (residues 5-381) was amplified from a human liver cDNA library and cloned into the pET-M vector (Novagen). The Hsc70 protein was expressed and purified as described previously [4].

Crystallization and Data Collection

All crystals were grown using sitting-drop vapor-diffusion. AtBAG5-long was crystallized by combining 1 μl of protein solution (18 mg/ml in 20 mM Tris-HCl pH 7.5, 500 mM NaCl, 1 mM DTT and 1 mM EDTA) with an equal volume of reservoir solution consisting of 1.5 M NaCl and 0.1 M NaAc (pH 4.5). Crystals were grown over one week at 20 °C and frozen in the cryoprotectant consisting of reservoir solution supplemented with 25% glycerol. AtBAG5-long/apo-CaM complex was crystallized by mixing 1 μl protein solution (15 mg/ml in 20 mM Tris-HCl pH 7.5, 500 mM NaCl, 1 mM DTT and 5 mM EDTA) with 1 μl reservoir buffer (containing 2.5 M ammonium sulfate and 0.1 M Tris-HCl, pH 8.5). Crystals were grown three days at 4 °C and frozen in the cryoprotectant of Paraton-N. The Se-Met-substituted crystals of AtBAG5-long/apo-CaM complex were produced as the wild type.

All data were collected on the BL19U1 beam line at Shanghai Synchrotron

Radiation Facility (SSRF) and processed using the HKL2000 software [5]. Single-wavelength anomalous data were collected for Se-Met-substituted crystals at the peak wavelength for Se.

Structure Determination and Refinement

The initial phase of AtBAG5-long structure was obtained by the program PHASER [6] using molecular replacement. Our previously solved AtBAG4 structure [4] was used as the searching model. Model was built using the program of COOT [7] and refined both by CNS [8] and PHENIX [9] programs. The noncrystallographic restraints were applied through the whole refinement. Iterative cycles of positional refinement were carried out until free R factor was converged.

The initial phases of AtBAG5-long/apo-CaM complex were obtained by molecular replacement using structures of AtBAG5-long, C- and N-terminal lobe of apo-CaM as searching models. The selenomethionines in CaM was used as a guide to locate the exact position of CaM molecules. The model was built manually using COOT program and refined using CNS program. The detailed data collection and refinement statistics are summarized in Table SI.

References

1 Clough, S. J. & Bent, A. F. Floral dip: a simplified method for Agrobacterium-mediated transformation of Arabidopsis thaliana. *Plant J* 16, 735-743 (1998).

2 Huber, P. A. *et al.* Multiple-sited interaction of caldesmon with Ca(2+)-calmodulin. *Biochem J* 316 (Pt 2), 413-420 (1996).

3 Maune, J. F., Klee, C. B. & Beckingham, K. Ca2+ binding and conformational change in two series of point mutations to the individual Ca(2+)-binding sites of calmodulin. *J Biol Chem* 267, 5286-5295 (1992).

4 Fang, S. S. *et al.* Structural insight into plant programmed cell death mediated by BAG

proteins in Arabidopsis thaliana. *Acta Crystallogr D Biol Crystallogr* 69, 934-945 (2013).

5 Otwinowski, Z., and Minor, W. Processing of X-ray diffraction data collected in oscillation mode. *Methods Enzymol* 276, 307-326 (1997).

6 McCoy, A. J. Solving structures of protein complexes by molecular replacement with Phaser. *Acta Crystallogr D Biol Crystallogr* 63, 32-41 (2007).

7 Emsley, P. & Cowtan, K. Coot: model-building tools for molecular graphics. *Acta Crystallogr D Biol Crystallogr* 60, 2126-2132 (2004).

8 Brunger, A. T. *et al.* Crystallography & NMR system: A new software suite for macromolecular structure determination. *Acta Crystallogr D Biol Crystallogr* 54, 905-921 (1998).

9 Zwart, P. H. *et al.* Automated structure solution with the PHENIX suite. *Methods Mol Biol* 426, 419-435 (2008).

Table SI Data collection and refinement statistics for AtBAG5 structures

**Crystal name**	**AtBAG5-long**	**AtBAG5-long/apo-CaM**
A. Data collection		
Space group	$\boldsymbol{P}6_222$	$\boldsymbol{P}2_12_12_1$
Unit cell (Å)	***a***= ***b***=78.79; ***c***=61.69	***a***=64.56; ***b***=74.89; ***c***=117.09
Wavelength (Å)	0.9795	0.9795
No. of unique reflections	7,844	19,469
Resolution range (Å)	50-2.0 (2.04-2.01)[a]	50-2.40 (2.49-2.40)[a]
Redundancy	20.1 (19.6)[a]	7.0 (7.3)[a]
$\boldsymbol{R}_{sym}$ (%)[c]	4.1 (35.7)[a]	8.3 (50.6)[a]
I/σ	73.1 (9.8)[a]	21.9 (5.3)[a]
Completeness (%)	99.9(100.0)[a]	99.6 (100.0)[a]
B. Refinement		
Resolution range (Å)	29.86-2.01	37.45-2.42
$\boldsymbol{R}_{crystal}$ (%)[d]	23.4	23.1
$\boldsymbol{R}_{free}$ (%)[e]	24.5	25.5
$RMSD_{bond}$(Å)	0.007	0.008
$RMSD_{angle}$(°)	1.200	1.255
Number of		
Proteins atoms	748	3,933
Solvent atoms	73	117
Residues in (%) regions		
Most favored	98.8	90.9
Additional allowed	1.2	8.4
Generously allowed	0	0.7
Disallowed	0	0
Average B factor ($Å^2$) of		
Chain A	45.05	57.96
Chain B		56.34
Chain F		64.75
Chain D		60.81
Solvent	56.05	57.73

[a] the highest resolution shell; [b] N_{obs}/N_{unique}; [c] $R_{sym} = \sum_j \left|I_j - \langle I \rangle\right| / \sum \langle I \rangle$ where $\boldsymbol{I}_j$ is the intensity of the jth reflection and $\langle I \rangle$s the average intensity.; [d] $\boldsymbol{R}_{crystal} = \sum_{hkl} \left|F_{obs} - F_{calc}\right| / \sum_{hkl} F_{obs}$; [e] $\boldsymbol{R}_{free}$ is calculated the same as $\boldsymbol{R}_{crystal}$, but from a test set containing 5% of data excluded from the refinement calculation.

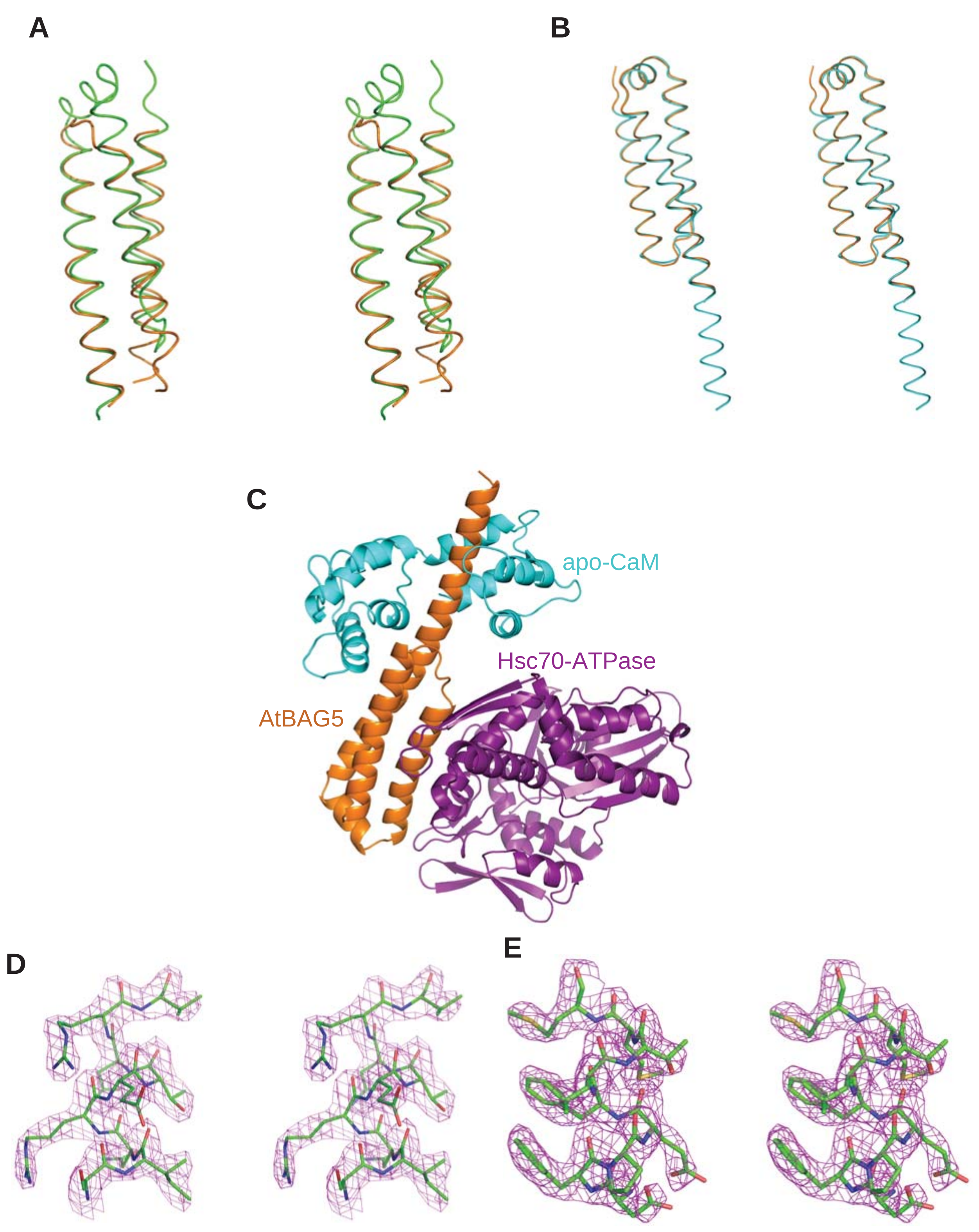

Figure S1 Structure of AtBAG5. (A) Stereoview of the superposition of AtBAG5-long (green) and AtBAG4 (orange, PDB code: 4HWH). **(B)** Stereoview of the superposition of AtBAG5-long alone (orange) and in complex with apo-CaM (cyan). **(C)** A model of the simultaneous interaction of AtBAG5 with apo-CaM and the Hsc70 ATPase domain. The BAG domains from the AtBAG5-long/apo-CaM and AtBAG1/Hsc70 complexes are superposed to simulate the interaction of AtBAG5 with apo-CaM and Hsc70. Represented 2***F***o-***F***c electron density map contoured at 1.5σ level for AtBAG5 alone **(D)** and in complex with apo-CaM **(E)**.

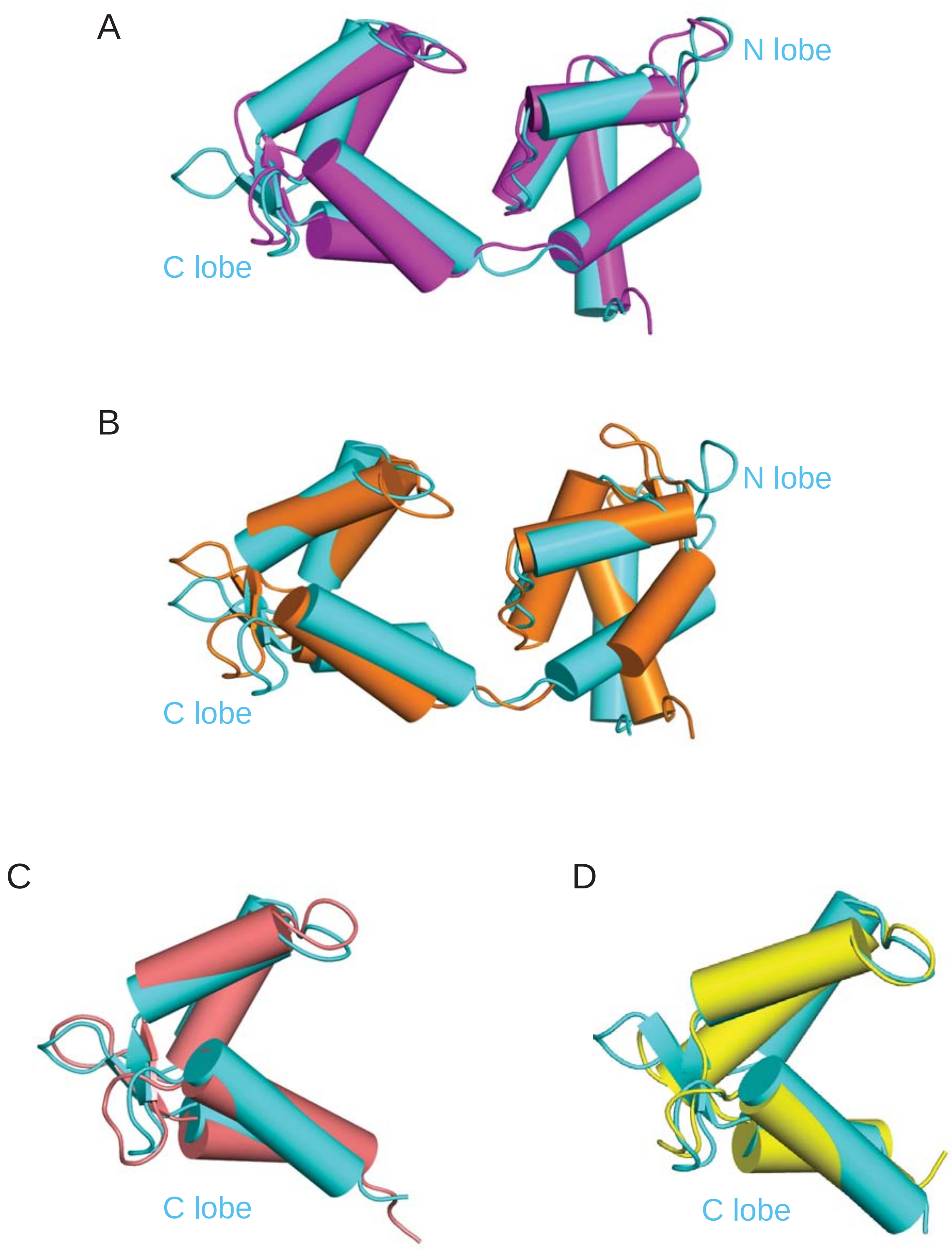

Figure S2 Superposition of the AtBAG5-long/apo-CaM complex with three reported IQ/apo-CaM complexes. (A) Superposition of apo-CaM in complex with AtBAG5-long (cyan) and apo-CaM in complex with the first IQ motif of myosin V (magenta; PDB ID: 2IX7). (B) Superposition of apo-CaM in complex with AtBAG5-long (cyan) and apo-CaM in complex with the second IQ motif of myosin V (orange). (C) Superposition of the C-lobe of apo-CaM in complex with AtBAG5-long (cyan) and that of apo-CaM in complex with the IQ motif of the $Na_V1.2$ voltage-dependent sodium channel (warm pink; PDB ID: 2KXW). (D) Superposition of the C-lobe of apo-CaM in complex with AtBAG5-long (cyan) and that of apo-CaM in complex with the IQ motif of the human cardiac sodium channel $Na_V1.5$ (yellow; PDB ID: 2L53).

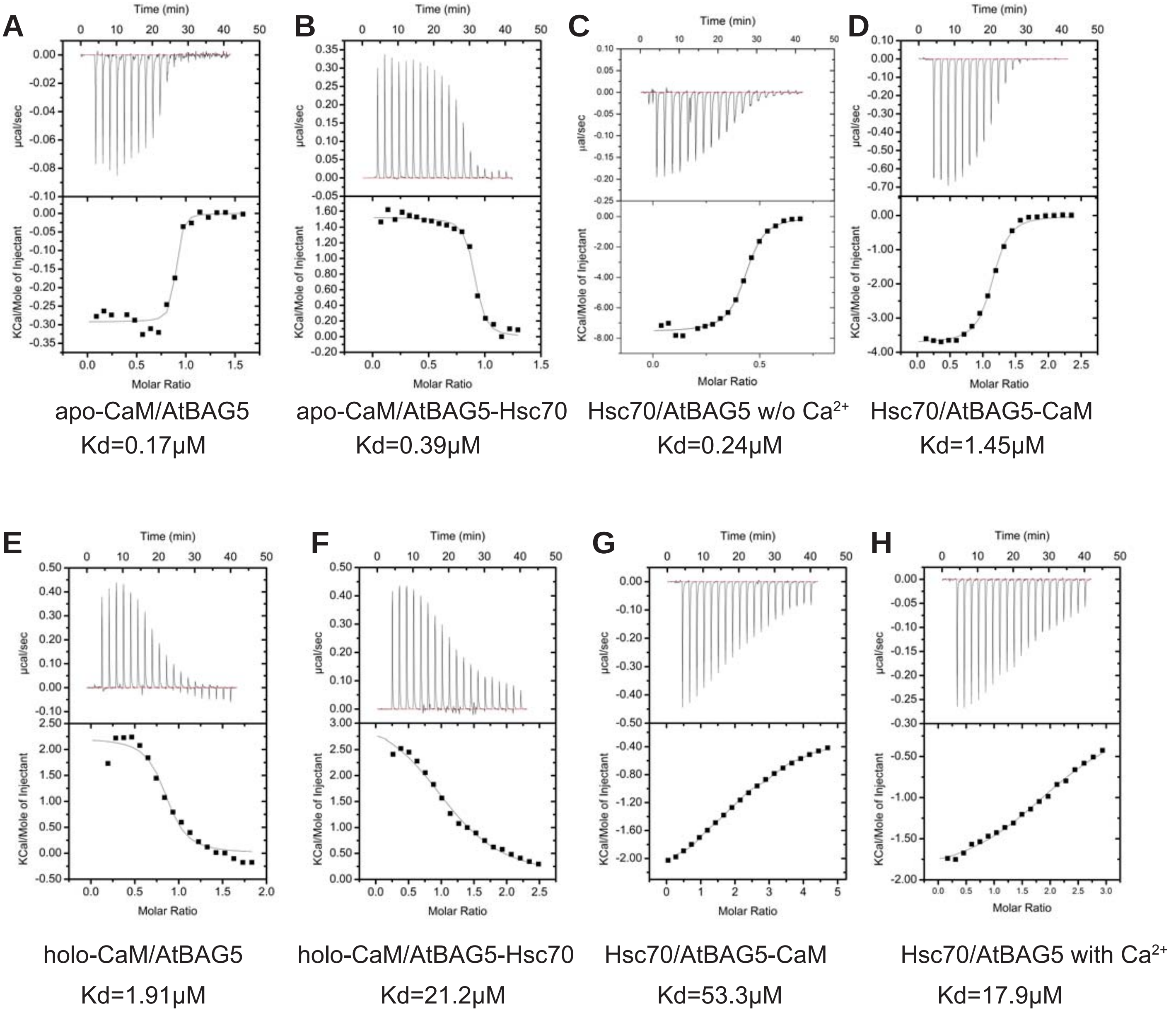

Figure S3 ITC analysis of the binding affinities among CaM, Hsc70 and AtBAG5-long. (A) ITC analysis of the binding affinity between apo-CaM and wild-type AtBAG5-long. **(B)** ITC analysis of the binding affinity between apo-CaM and the complex of AtBAG5-Hsc70 ATPase domain. **(C)** ITC analysis of the binding affinity between the Hsc70 ATPase domain and AtBAG5-long in the absence of Ca^{2+}. **(D)** ITC analysis of the binding affinity between the Hsc70 ATPase domain and the complex of AtBAG5-CaM in the absence of Ca^{2+}. **(E)** ITC analysis of the binding affinity between holo-CaM and wild-type AtBAG5-long. **(F)** ITC analysis of the binding affinity between holo-CaM and the complex of AtBAG5-Hsc70 ATPase domain. **(G)** ITC analysis of the binding affinity between the Hsc70 ATPase domain and AtBAG5-CaM in the presence of Ca^{2+}. **(H)** ITC analysis of the binding affinity between the Hsc70 ATPase domain and the AtBAG5-long in the presence of Ca^{2+}.

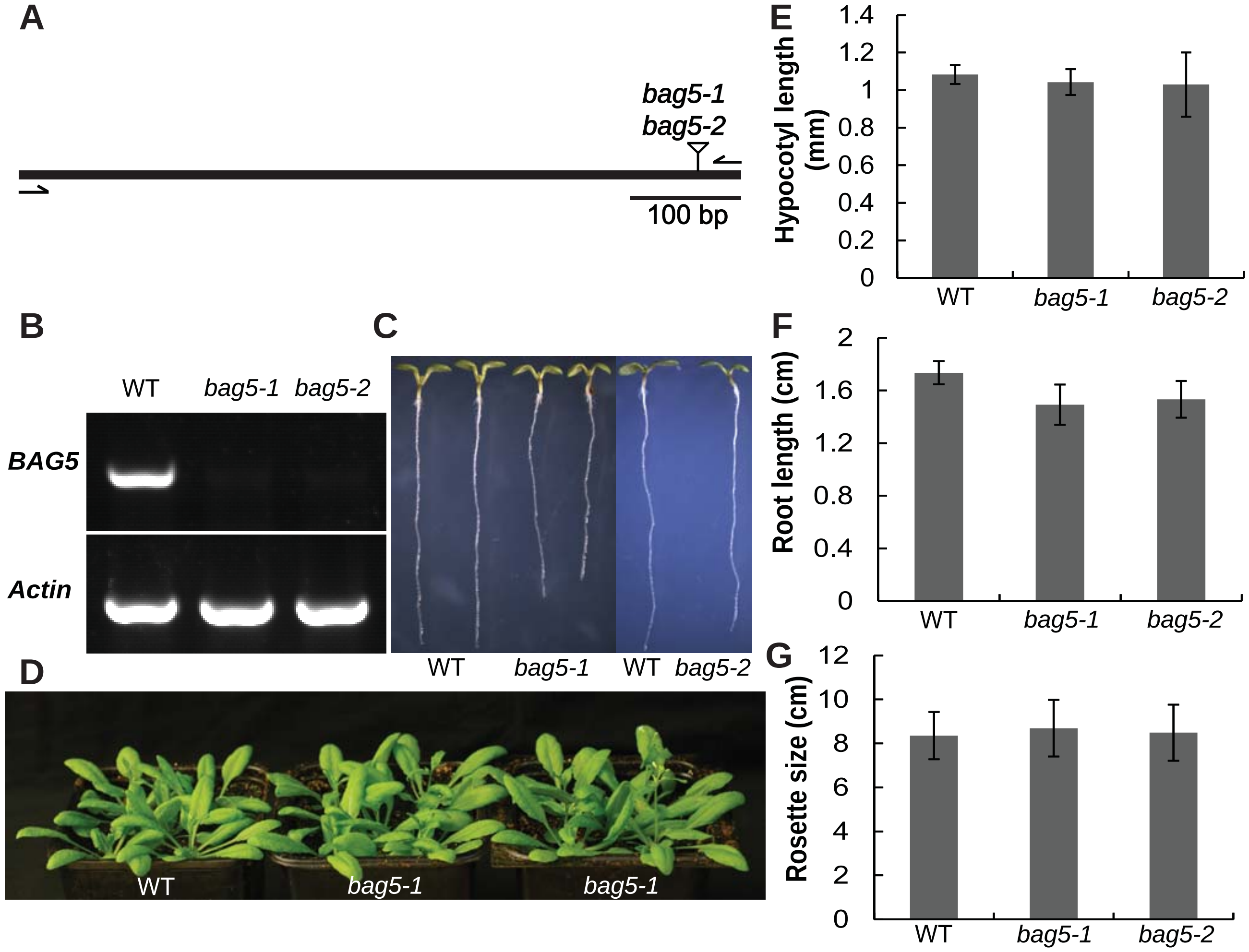

Figure S4 Isolation and characterization of the *bag5-1* and *bag5-2* mutants. (A) Schematic representation of T-DNA insertion into the exon of the AtBAG5 locus (AT1G12060; triangle). Thick black lines represent exons, and arrows represent RT-PCR primers; the scale bar corresponds to 100 bp. **(B)** Semi-quantitative RT-PCR analysis of wild-type (wt) and the *bag5-1/bag5-2* mutants; the *Actin* gene was used as a reference. **(C)** Comparison of 6-day-old wt and *bag5-1/bag5-2* seedlings. **(D)** Comparison of 4-week-old wt and *bag5-1/bag5-2* plants. **(E, F)** Quantitative analyses of the hypocotyl length and root length of 6-day-old wt and *bag5-1/bag5-2* mutant seedlings; average ± SD, n = 17. **(G)** Quantitative analyses of the rosette size of 4-week-old wt and *bag5-1/bag5-2* plants; average ± SD, n ≥ 17.

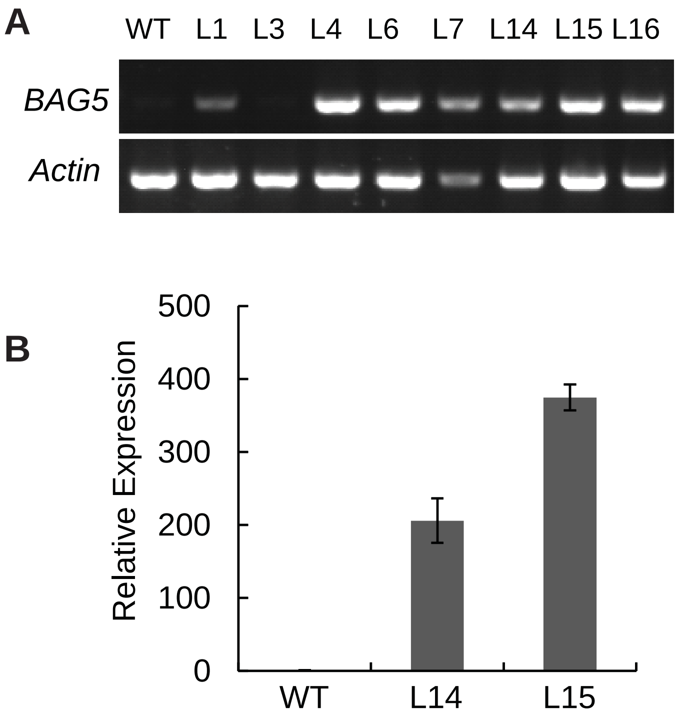

Figure S5 Analysis of *AtBAG5* expression levels in *OxBAG5* transgenic plants. (A) RT-PCR analysis of *AtBAG5* transcript levels in *OxBAG5* transgenic lines. **(B)** Quantitative RT-PCR analysis of Wild type, *OxBAG5* transgenic lines L14 and L15. Means were calculated from two biological samples, and each biological sample was examined in triplicate.

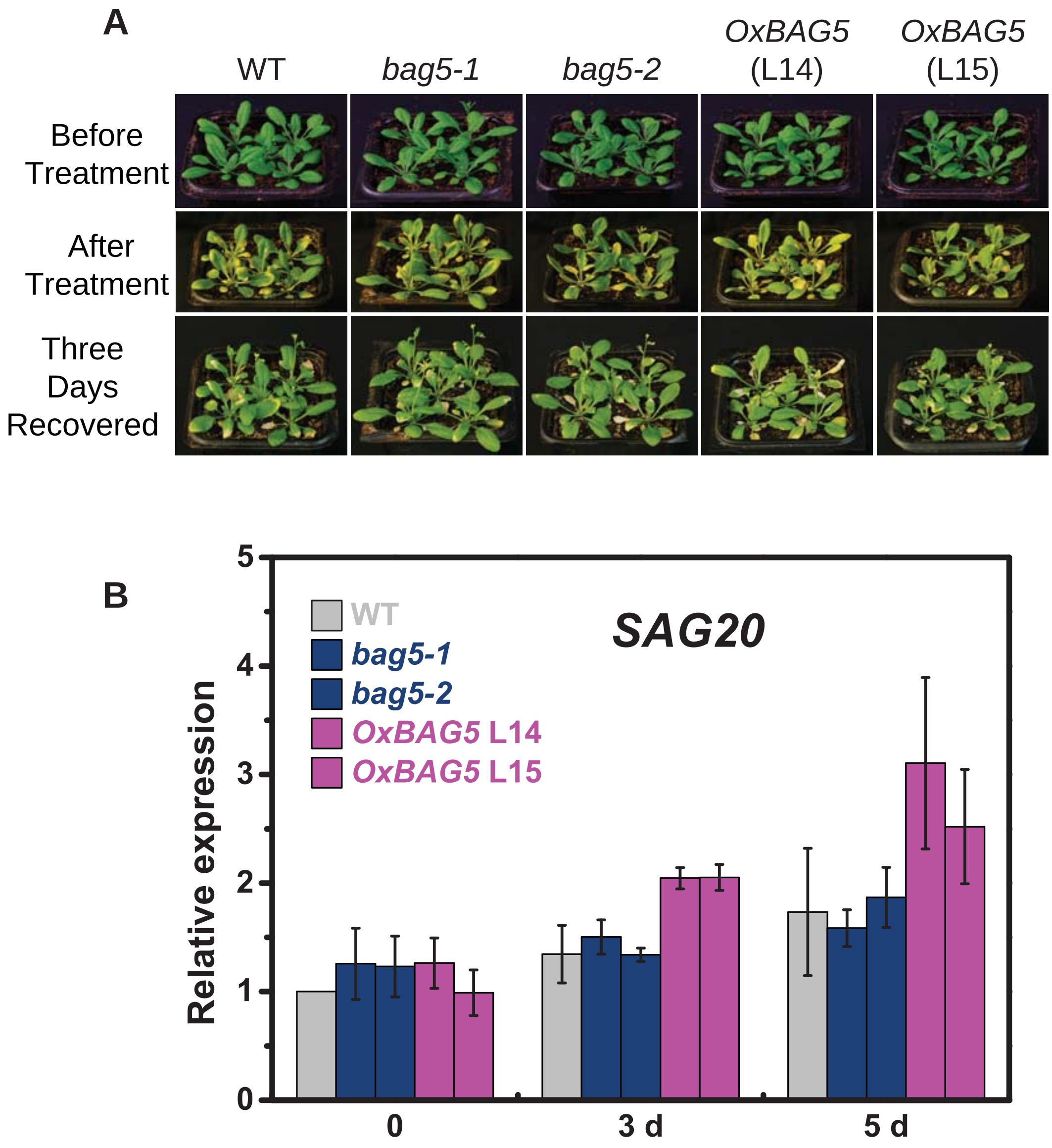

Figure S6 Effect of AtBAG5 overexpression on leaf senescence. (A) Twenty-four-day-old plants were treated with total darkness and then returned to normal light growth conditions to recover. The images were taken prior to treatment, after treatment and after three days of recovery. The experiment was performed with two replicates. **(B)** The expression levels of *SAG20* in the wild type, mutants, and overexpression lines. Values are mean ± SE (n = 3 experiments).
